# Deep brain stimulation of the anterior nucleus of the thalamus for seizures after new-onset refractory status epilepticus: a case report

**DOI:** 10.3389/fnhum.2025.1663280

**Published:** 2025-10-31

**Authors:** Ryota Sasaki, Hiroya Ohara, Masako Kinoshita, Takahiro Iizuka, Kentaro Tamura, Kiyoshi Nagata, Ichiro Nakagawa

**Affiliations:** ^1^Department of Neurosurgery, Nara Medical University, Kashihara, Japan; ^2^Department of Neurosurgery, National Hospital Organization Nara Medical Center, Nara, Japan; ^3^Department of Neurology, Minaminara General Medical Center, Yoshino, Japan; ^4^Department of Neurology, National Hospital Organization Utano National Hospital, Kyoto, Japan; ^5^Department of Neurology, Kitasato University School of Medicine, Sagamihara, Japan

**Keywords:** new-onset refractory status epilepticus, NORSE, anterior nucleus of thalamus deep brain stimulation, DBS, drug-resistant epilepsy, immunotherapy, video-electroencephalogram monitoring, temporal lobe epilepsy

## Abstract

**Objectives:**

Most patients with new-onset refractory status epilepticus (NORSE) subsequently develop drug-resistant epilepsy (DRE) with multiple seizure foci and are not the typical candidates for resective surgery. We report the first case of DRE developing after cryptogenic-NORSE (C-NORSE) that was successfully treated using deep brain stimulation targeting the anterior nucleus of the thalamus (ANT-DBS).

**Methods:**

A 52-year-old man developed C-NORSE at the age of 45 years and presented with sequelae of DRE and cognitive dysfunction despite anti-seizure medications and immunotherapy administration. Seizure semiology comprised palpitations, chills, and nausea, followed by impairment of awareness with oral automatism multiple times a day. Video-electroencephalogram monitoring (vEEG) showed bilateral independent electrographic seizures (ESz) in the fronto-temporal areas. He underwent ANT-DBS. Preoperative and postoperative vEEG recordings for 3 days were compared.

**Results:**

Preoperative vEEG showed 11 clinical seizures correlated with ESz. The duration of ESz ranged from 55 to 213 s (median, 81 s). Three months after ANT-DBS stimulation, vEEG showed four subclinical ESz episodes lasting from 22 to 31 s (median, 25.5 s) (Mann–Whitney *U* test, *p* = 0.001). The patient had not developed an overt clinical seizure until the last follow-up at 9 months. No adverse events were observed during treatment.

**Conclusion:**

ANT-DBS is an effective treatment option for DRE after NORSE, particularly when the epileptogenic network is located in the temporal lobe. A detailed evaluation using vEEG is useful for identifying the epileptogenic foci and assessing therapeutic outcome. Immunomodulatory mechanisms via cytokines could play roles in the pathogenesis of development of DRE after NORSE and seizure suppression effect of ANT-DBS.

## Introduction

New-onset refractory status epilepticus occurs in patients with no history of epilepsy or other neurological disease and manifests as persistent refractory epileptic seizures without any evidence of structural, toxic, or metabolic etiologies in the acute phase (Bhatia and De Jesus, 2025). The term new-onset refractory status epilepticus (NORSE) was first used by Wilder-Smith et al. (2005), and a consensus definition of NORSE and related diseases was proposed in 2018 to improve patient management and clinical research (Hirsch et al., 2018). When its cause remains unknown after extensive workup, it is called as cryptogenic NORSE (C-NORSE) (Bhatia and De Jesus, 2025). NORSE presents as refractory status epilepticus and leads to death in 22% of adult patients (Gaspard et al., 2015). Almost all survivors develop drug-resistant epilepsy (DRE) and severe cognitive dysfunction. The common seizure types are focal impaired awareness seizures (FIAS) and focal aware seizures (FAS) (Kramer et al., 2011), and most patients have multiple seizure foci. These patients are not the typical candidates for resective surgery.

The anterior nucleus of the thalamus (ANT) is the most commonly studied target in deep brain stimulation (DBS) for DRE. ANT-DBS is effective for focal epilepsy, with a reported seizure reduction of approximately 75% after 10-year follow-up (Salanova et al., 2021). In Japan, regulatory approval was granted to ANT-DBS for DRE in December 2023.

In this study, we report a case of medically intractable bilateral temporal lobe epilepsy after C-NORSE who was successfully treated using ANT-DBS. The therapeutic effect of ANT-DBS was investigated using video-electroencephalogram monitoring (vEEG), which demonstrated statistically significant improvement of epileptic seizures.

## Case description

**Figure 1:**
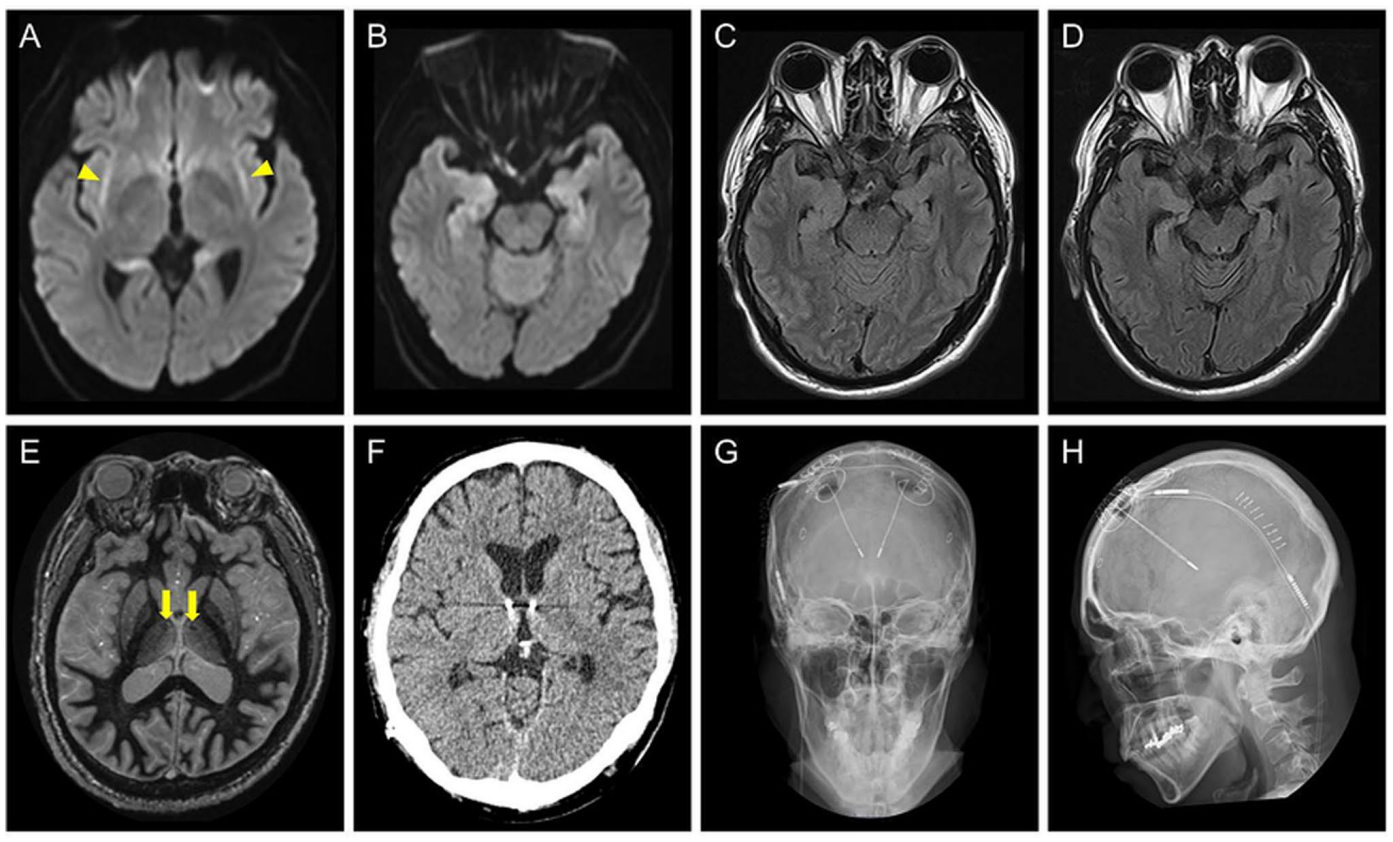
**(A–C)** Brain axial MRI of the patient at the age of 45 years, during the acute phase of cryptogenic new-onset refractory status epilepticus. Diffusion-weighted images show bilateral claustrum signs (**A**, yellow arrowheads) and bilateral hippocampal abnormalities **(B)**. A fluid attenuated inversion recovery (FLAIR) image shows high signal intensity areas in the hippocampi **(C)**. **(D)** The axial FLAIR image at the age of 51 years showing progression of atrophy of the bilateral hippocampi. **(E)** A preoperative fast gray matter acquisition T1 inversion recovery sequence at the age of 52 years shows the intact mammillothalamic tract (yellow arrows). **(F–H)** Postoperative brain axial CT **(F)** and skull radiographic **(G,H)** images showing placement of the stimulation leads.

A 52-year-old right-handed man with DRE visited our hospital for comprehensive preoperative assessment of epilepsy. At the age of 45 years, he developed C-NORSE, which required intensive treatment, including intubation. Cerebrospinal fluid (CSF) analysis revealed 12 white blood cells/μL and normal protein and glucose levels. Magnetic resonance imaging (MRI) during the acute phase showed bilateral claustrum signs and mesial temporal abnormalities (Figures 1A–C). An autoimmune mechanism was initially suspected and extensively examined at the laboratory of Josep Dalmau (Barcelona) through Kitasato University for potential autoantibodies against neuronal surface antigens, including NMDA, AMPA, GABA(A), GABA(B), mGluR1, and mGluR5 receptors, LGI1, Caspr2, DPPX, Neurexin3, and Iglon5, with established assay using in-house immunohistochemistry and cell-based assay; however, no neuronal surface autoantibodies were identified in CSF. The patient was finally diagnosed with C-NORSE and was treated with numerous anti-seizure medications (ASM), corticosteroids, and high-dose immunoglobulins; despite this, he ultimately developed DRE. No other relevant medical or family history was found. The patient was administered lacosamide 400 mg, perampanel 8 mg, levetiracetam 2,500 mg, lamotrigine 175 mg, valproic acid 600 mg, and prednisolone 25 mg.

The seizure semiology comprised FAS with palpitations, chills, and nausea, followed by FIAS with oral automatism multiple times a day. Neurological examination findings were unremarkable except for cognitive dysfunction. Assessment using the Wechsler Adult Intelligence Scale-4th edition revealed low intelligence scores (Full Scale Intelligence Quotient 64, Verbal Comprehension Index 77, Perceptual Reasoning Index 69, Working Memory Index 71, Processing Speed Index 66, and General Ability Index, 70). MRI showed bilateral hippocampal atrophy, whereas the mammillothalamic tract was intact (Figures 1D,E). vEEG showed bilateral independent spikes and electrographic seizures (ESz) arising from the fronto-temporal areas. Based on the diagnosis of DRE with multiple seizure foci in the bilateral mesial temporal lobes, the patient underwent ANT-DBS (Figures 1F–H). Stimulation was started on the 7th postoperative day with the following parameters: 1 mA (gradually increased to 1.5 mA and then 1.8 mA every 6 weeks), 145 pulses/s, 90 μs, 1 min on, and 5 min off.

To evaluate the effect of our intervention on the suppression of epileptic seizures, vEEG recordings were compared before and after treatment.

This study was conducted in accordance with the principles of the Declaration of Helsinki, and the case report was prepared in compliance with the CARE guidelines. Approval of the study design was waived by the Ethics Committee of Nara Medical University based on its classification as a retrospective analysis of anonymized clinical data. Written informed consent was obtained from the patient.

## Diagnostic assessment

vEEG was performed for 3 days pre- and postoperatively using EEG-1200 (Nihon-Kohden Tokyo, Japan) via scalp electrodes placed according to the International 10–20 system with T1/T2. The EEG data were retrospectively reviewed by two board-certified epileptologists (RS and HO) using a longitudinal bipolar montage with T1–T2 and A1–A2 derivations. ESz and electroclinical seizures that satisfied the criteria of the American Clinical Neurophysiology Society’s Standardized Critical Care EEG Terminology: 2021 Version (Hirsch et al., 2021) were identified. Seizure semiology was confirmed using video recording and patient interview. The number and duration of seizures were analyzed. As the data were not normally distributed, the Mann–Whitney *U* test was employed to evaluate statistical significance. The significance level was set at *p* = 0.05. All statistical analyses were performed using SPSS Statistics 27.0J software (IBM Japan, Tokyo, Japan).

**Figure 2:**
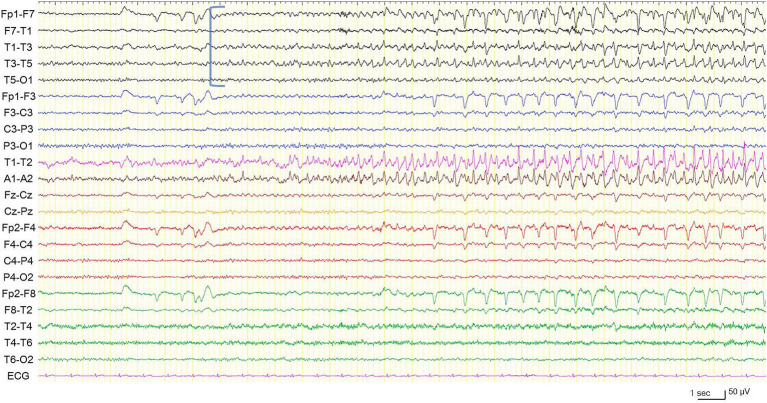
Electroencephalogram during a habitual focal impaired awareness seizure. Electrographic seizure is arising from the left temporal region (blue square bracket). High frequency filter 60 Hz, time constant 0.1 s.

**Figure 3:**
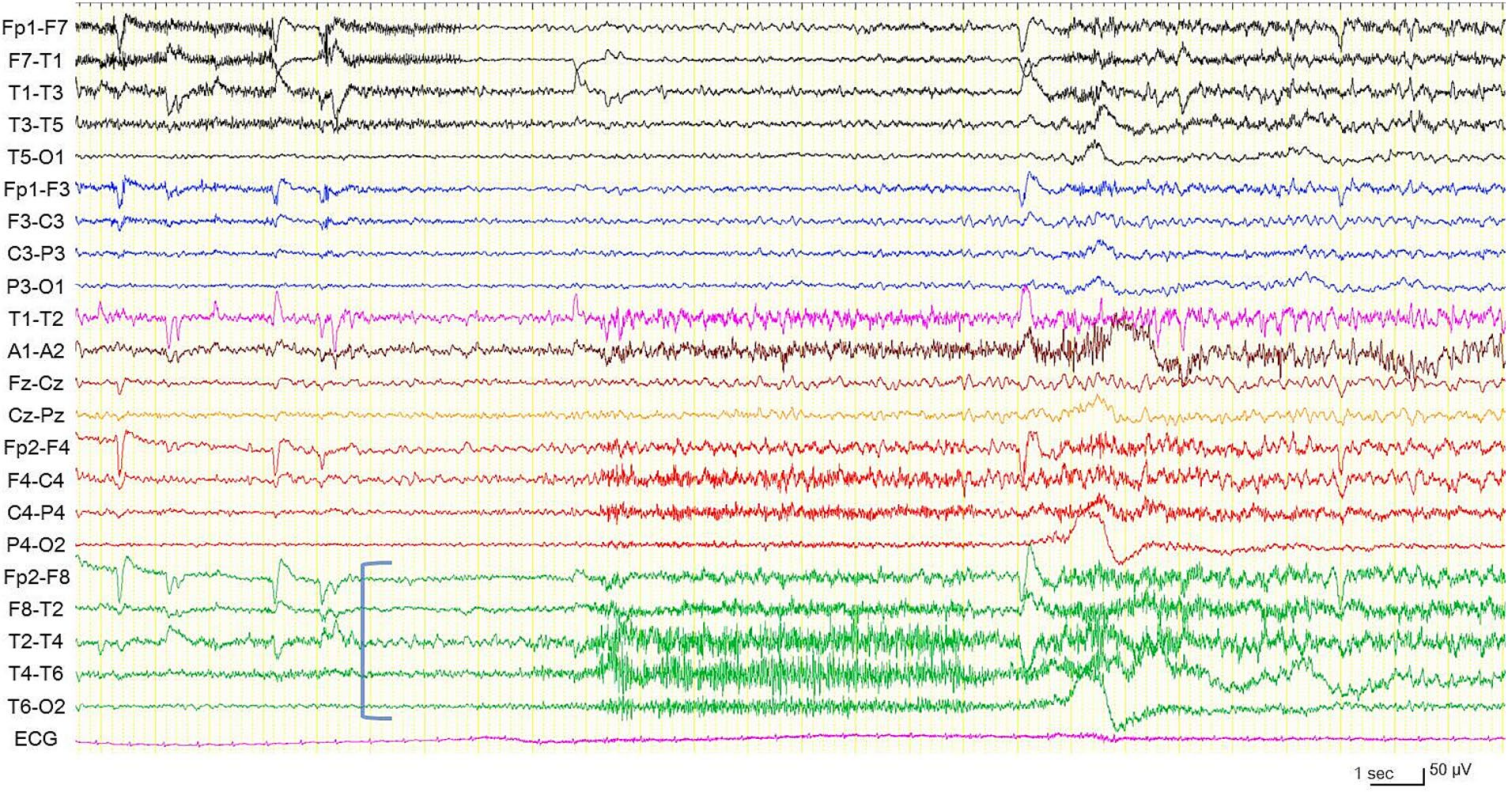
Electroencephalogram during another habitual focal impaired awareness seizure, different from the one shown in Figure 2. Electrographic seizure arising from the right temporal region (blue square bracket). High frequency filter 60 Hz, time constant 0.1 s.

In preoperative vEEG, 11 habitual FIAS correlated with ESz. All ESz arose from the fronto-temporal areas: eight on the left and three on the right (Figures 2, 3). The duration of ESz ranged from 55 to 213 s (median, 81 s). Three months after postoperative stimulation, vEEG showed four subclinical ESz episodes lasting from 22 to 31 s (median, 25.5 s), three on the left, and one on the right. No clinical seizures were observed. The duration of ESz significantly decreased after ANT-DBS (*p* = 0.001). The dose of prednisolone was reduced to 10 mg, and the patient had not developed an overt clinical seizure until the last follow-up at 9 months. No adverse events were observed during treatment. Interleukin (IL) levels were not examined.

## Discussion

To the best of our knowledge, this is the first reported case of DRE developing after C-NORSE who was successfully treated with ANT-DBS. In this case, ANT-DBS was selected after careful consideration of surgical indications. After treatment, clinical seizures improved, and the prednisolone dose was reduced. Moreover, using vEEG analyses, we documented significant EEG changes in the number and duration of ESz.

Most cases of epilepsy after NORSE and its subcategory, febrile-infection related epilepsy syndrome (FIRES), are drug-resistant and often require multiple ASM, immunomodulation, and immunosuppressive therapies. Surgical treatment can be considered, but multiple seizure foci involving extensive epileptogenic networks often hinder focal resective surgery. Therefore, neuromodulation therapies are performed as palliative surgery (Oliger et al., 2024). In our case, seizure semiology and imaging and EEG findings clearly indicated that seizures originated within the limbic system, and ANT-DBS was expected to be effective for seizure suppression as it is effective in intractable temporal lobe epilepsy (Fisher et al., 2010; Yan et al., 2023). A controlled clinical trial of ANT-DBS (SANTE study) showed initial seizure reduction by 20–30% in the control group until three postoperative months, possibly due to the microlesion effect (Fisher et al., 2010). However, the effect in our patient was complete suppression of disabling clinical seizures for more than 3 months. Besides treatment for DRE, ATN-DBS has been utilized to control refractory and super-refractory status epilepticus (Lee et al., 2017; Imbach et al., 2019; Yuan et al., 2019; Sobstyl et al., 2020). Yuan et al. (2019) reported a patient who showed successful control of super-refractory status epilepticus with ANT-DBS for 2 years but died from refractory convulsive status epilepticus after removal of the stimulator. Other targets can be selected in accord with seizure types. Previous studies described that DBS targeting the centromedian thalamic nucleus was effective in suppressing super-refractory status epilepticus, especially generalized convulsions, including four patients with acute-phase NORSE and FIRES within 2 months from the onset (Valentín et al., 2012; Lehtimäki et al., 2017; Sa et al., 2019; Sobstyl et al., 2020; Stavropoulos et al., 2021; Hect et al., 2022; Stavropoulos et al., 2023). Low frequency centromedian thalamic nuclei DBS (6 pulses/s, 300 μs) has been reported to reduce focal/multifocal seizures subsequent to improvement of generalized convulsions (Valentín et al., 2012; Sa et al., 2019; Stavropoulos et al., 2021). Considering the high mortality and seizure recurrence rate for refractory status epilepticus (Kämppi et al., 2024; Lattanzi et al., 2024; Gettings et al., 2025), further advancements in acute-phase management could improve the prognosis and prevent intractable epilepsy after NORSE.

In general, ANT-DBS is more effective for DRE than vagus nerve stimulation (VNS) (Salanova et al., 2021; Fisher et al., 2010). One patient with FIRES, presenting with persistent status epilepticus despite pharmacologic management, immunotherapy, and VNS, showed considerable seizure reduction and cognitive function improvement after centromedian thalamic nucleus DBS (Hect et al., 2022; Stavropoulos et al., 2023). A recent review summarized 15 patients with NORSE treated with VNS and reported that three out of 12 patients achieved complete freedom from seizures in the long-term follow-up (range, 166 days to 1 year in seizure-free patients) (Mantoan Ritter and Selway, 2023). A case series of three patients who underwent responsive neurostimulation after NORSE described that all patients showed a modest improvement in seizure frequency and severity, but two of them continued to have relatively disabling seizures (Oliger et al., 2024).

While there was no side effect in our patient, neuropsychiatric adverse events are reported during long-term follow-up of ATN-DBS. Salanova et al. (2021) observed depression in 37.3%, memory impairment in 27.3%, and suicidal ideation in 11.8% out of 110 patients during five-year follow-up; however, 66% of the patients reporting depression had a history of depression and 50% of the patients reporting memory impairment had a history of memory impairment. Järvenpää et al. (2018) reported two patients with a history of depression who showed sudden depressive symptoms related to DBS, which were ameliorated by reducing the stimulation voltage and changing the electrode contacts. Therefore, special caution is required to consider and maintain ATN-DBS in DRE patients with a history of these conditions. As chronic pain closely associated with psychological distress and sleep problems, device-related adverse events such as local pain, paresthesias, and discomfort can aggravate depression, cognitive impairment, and anxiety, leading to social withdrawal and isolation (Salanova et al., 2021; Cohen et al., 2021). Changes in therapy may have a negative impact on the emotional well-being of patients with epilepsy mainly due to anxiety concerning to therapeutic effect and additional seizure activities (Fishman et al., 2017); changes in seizure types from FIAS to FAS could force patients to recognize their seizure symptoms. On the other hand, neuropsychological test scores showed statistically significant improvement in attention, executive function, depression, tension/anxiety, total mood disturbance, and subjective cognitive function (Salanova et al., 2021). Another study showed a statistically significant improvement in delayed verbal memory more than 1 year (mean, 15.9 months) after ATN-DBS surgery, presumably associated with the activation of the fronto-limbic circuit (Oh et al., 2012). These findings are in concordant with postoperative cognitive improvement, associated with increased glucose metabolism in the dorsolateral prefrontal cortex and in the dorsomedial and ventromedial frontal cortices, in mesial temporal lobe epilepsy after subtemporal amygdalohippocampectomy (Takaya et al., 2009).

The mechanisms by which ANT-DBS suppresses epileptic seizures are not fully understood. The positive effects in temporal lobe epilepsy can be associated with the anatomical reasoning that both ANT and mesial temporal structures are components of the limbic system (Fisher et al., 2010). A one-year follow-up study on changes in plasma IL-6, a proinflammatory, pro-convulsive, and neurotoxic cytokine, and IL-10, an anti-inflammatory and neuroprotective cytokine, in 22 patients with DRE who underwent ANT-DBS showed that the IL-6/IL-10 ratio before DBS was higher in responders than in non-responders, and the ratio significantly decreased over time following DBS in the whole group (Basnyat et al., 2021). IL-6 elevation in the plasma and CSF has been reported in cases of NORSE (Sakuma et al., 2015). Though we could not evaluate the cytokine levels in our patient, immunomodulatory mechanisms via cytokines could play roles in the pathogenesis of development of DRE after NORSE and seizure suppression effect of ANT-DBS.

This case study has three main limitations. First, as a single case without a depressive episode was analyzed in this study, we could not know the effect on patients with a various background, especially with a history of depression. Second, the follow-up length is less than 1 year and long-term outcome have not been evaluated. Third, data on ILs and other cytokines could not be obtained. Further studies are warranted to address these concerns.

In summary, ANT-DBS is a good treatment option for DRE developing after NORSE, particularly when the epileptogenic network resides in the limbic system, including the mesial temporal lobe. A detailed evaluation using vEEG is useful for identifying the epileptogenic foci and assessing therapeutic outcome.

## Patient perspective

The present observations indicate that ANT-DBS is an effective treatment option for DRE after NORSE, particularly when the epileptogenic network is located in the limbic system including the temporal lobe. A detailed evaluation using vEEG is useful for identifying the epileptogenic foci and assessing therapeutic outcome. Immunomodulatory mechanisms via cytokines could play roles in the pathogenesis of development of DRE after NORSE and seizure suppression effect of ANT-DBS.

## Data Availability

The original contributions presented in the study are included in the article/supplementary material, further inquiries can be directed to the corresponding author.
